# Fabrication of nanozyme thixotropic anionic hydrogel for treating fungal keratitis by Dectin-1/p38 pathway

**DOI:** 10.1007/s00253-025-13529-8

**Published:** 2025-06-26

**Authors:** Chenchen Zhang, Jia Li, Xinyue Shen, Jihong Wang

**Affiliations:** Department of Ophthalmology, Affiliated Hospital of Jiangnan University, Wuxi, No. 1000, Hefeng Road, 214122 China

**Keywords:** Fungal keratitis, Anionic hydrogel, Itraconazole, Anti-inflammation

## Abstract

**Abstract:**

Fungal keratitis (FK) is a major cause of corneal blindness, particularly in China, where treatment is often limited by systemic side effects and antifungal drug resistance. We propose a nanozyme (carbon nanotube (CNT))-based anionic hydrogel coating (NHC) loaded with itraconazole (IZ) (NTH@CNT/IZ) as a treatment for FK to overcome these challenges. This formulation was designed to enhance ocular drug delivery, improve antifungal efficacy, and reduce inflammation. In vitro assays against *Aspergillus fumigatus* demonstrated potent antifungal activity, including significant reductions in colony-forming units and biofilm formation at 50 µg/mL. Cell viability tests using ARPE-19 cells revealed high biocompatibility, with no observed morphological alterations or apoptosis, despite increased ROS and DNA proliferative activity. Importantly, NTH@CNT/IZ markedly downregulated inflammatory mediators—Dectin-1, IL-1β, and TNF-α—and inhibited phosphorylation of p38 MAPK, indicating suppression of the Dectin-1/p38 MAPK signaling pathway. In vivo results further confirmed its therapeutic potential, showing reduced corneal fungal burden and inflammation, along with effective penetration through the corneal epithelium, overcoming mucosal and fungal barriers. Together, these findings highlight NTH@CNT/IZ as a promising localized treatment strategy for fungal keratitis, offering targeted antifungal action and immune modulation to improve clinical outcomes and preserve ocular integrity.

**Key points:**

- *Nanozyme hydrogel for ocular health*
- *In vitro antifungal efficacy*

**Supplementary Information:**

The online version contains supplementary material available at 10.1007/s00253-025-13529-8.

## Introduction

Fungal keratitis (FK) is the most common eye infection that leads to blindness and other eye damage. *Fusarium* and *Aspergillus* were the main fungi that caused fungal keratitis (Manikandan et al. 2019). Bacterial keratitis has the highest prevalence rate among infectious keratitis, which can be caused by fungi, viruses, or parasites. Infectious keratitis is the most common cause of corneal ulcers seen in clinics (Cheng et al. 2022). With 26% of cases in the UK, 30% in the northern USA, 41% in the Middle East, and 18–60% in Asia, *Aspergillus* stands out among the pathogenic fungi that cause fungal keratitis. According to reports, keratoplasty is necessary for 42.60% of patients with *Aspergillus keratitis* (Acar et al. 2011). *Aspergillus*, *Candida*, and *Fusarium* are the most common genera that cause infections. The initial FDA-approved treatment standard for fungal keratitis was a topical natamycin suspension (5%) (Sathe et al. 2024). The fungus *A. fumigatus* is the most common cause of *Aspergillus keratitis* worldwide, regardless of economic status. Keratitis caused by *A. fumigatus* is more clinically significant, has a higher recurrence rate, and has a worse prognosis than that of *Aspergillus flavus* (Botterel et al. 2008). Corneal ulcers, second only to cataracts, are the leading cause of monocular blindness and visual impairment in many developing countries. Fungal invasiveness, virulence factor production, and biofilm formation significantly contributed to corneal damage, along with excessive inflammation (Plummer 2017). Natamycin and voriconazole are two examples of conventional antifungal medications, but their lack of anti-inflammatory effects, poor solubility, and instability prevent them from providing optimal therapeutic efficacy (Liu et al. 2013). Itraconazole is used to treat serious fungal or yeast infections (Boucherit-Atmani et al. 2011). Itraconazole and caspofungin (CAS) are two antifungal medications that are currently used to treat invasive *Aspergillosis* (Saremi et al. 2024). Although antimicrobial and anti-inflammatory effects have received a lot of attention, little is known about the non-antifungal effects. The potential mechanisms of action of itraconazole and its potential non-antifungal uses in dermatology were described (Tsai and Tsai 2019). Itraconazole and the carrier were combined to create a new solution formulation that is now being tested in clinical trials for its improved absorption (Liu et al. 2013). Hence, it is imperative to contemplate the investigation of a more potent pharmaceutical agent that possesses both anti-inflammatory and antifungal attributes. This effect was achieved by activating the Nrf2/HO1 signaling pathway (Sahay et al. 2019). In the field of colloid science, thixotropy is among the earliest rheological phenomena that have been recorded. The hydrogel-based NPs produce a solution, but the sol–gel transition happens when the mixture is left to rest (Zanna and Tomasini 2017). Carbon nanotubes (CNTs) are cylindrical nanostructures made from graphene sheets rolled into tubes. They possess unique properties, including high surface area, excellent electrical conductivity, and strong mechanical strength, making them valuable for various applications in nanotechnology. One area where CNTs have proven particularly effective is in adsorption processes, where contaminants are removed from liquids or gases by attaching to the surface of a material. CNTs are a versatile, highly effective adsorbent in nanotechnology for water treatment applications, offering significant improvements over traditional methods in terms of adsorption capacity, reusability, and overall efficiency in removing complex contaminants (Musere et al. 2021). Using hydrogel-incorporated carbon nanotubes for drug delivery can improve the sustained effect of dual-stage release and enable controlled light irradiation for drug delivery (Dong et al. 2017). Regarding drug delivery, carbon nanotube–thermoresponsive hydrogels have a distinct advantage. Bio-nanocomposite hydrogels can be reinforced with a wide variety of functional materials, including nanoclays, cellulose nanocrystals, graphene oxide, carbon nanotubes (CNTs), fibrin, fibronectin, nanocollagen, and cellulose nanowhiskers (Ravanbakhsh et al. 2020). The number of guanosine molecules controls the number of hydrogen bonds throughout the system, which gives the final hydrogel its thixotropic and injectable properties (Sardaru et al. 2021) (Jain and Matsumura 2016). Until our prior research on an LMWG/inorganic nanosheet system, no one has documented the use of a synthetic gelator in conjunction with an inorganic nanosheet to create a thixotropic gel to treat infectious diseases. The research on this topic has detailed easy ways to make hydrogels out of magnetic composite polymers composed of iron oxide particles and polyvinyl groups (Ohsedo et al. 2017). The synthesis and biological applications of a few exemplary thixotropic hydrogels are explored, with particular emphasis on their use in encapsulation and cell culture (Pramanik 2022). By secreting IL-1β and IL-6, transformed human corneal epithelial cells (HCECs) responded to challenge with TLR2 and TLR4 ligands. Moreover, they started an intrinsic immune response and identified *A. fumigatus* hyphae using TLR2 and TLR4 receptors (Dai et al. 2019). The type of fungi with different types of materials has a particular role in fungal keratitis, which is listed in Table 1. Based on antifungal and anti-inflammatory action, oxymatrine (OMT) has a curative effect on *A. fumigatus* keratitis. However, there is currently no research on the possible mechanism of OMT (Liu et al. 2024). *A. fumigatus* is the most often occurring airborne fungus since the recent rise in immunosuppressive treatments leaves more of the population susceptible to infection (Cruz and Wuest 2023). Animal models show that quercetin reduces *A. fumigatus* keratitis by blocking TLR4. The mechanism by which quercetin prevents A. fumigatus keratitis in macrophages involves the inhibition of inflammatory signaling pathways (Luan et al. 2023). The Dectin-1 receptor is a key player in the process of β-glucan recognition on APCs, which encompass a fraction of T cells, macrophages, dendritic cells, monocytes, and neutrophils (Mochizuki and Sakurai 2011). The eugenol protects *A. fumigatus* keratitis which is unknown from damage. The possible mechanism of eugenol was investigated to determine its anti-inflammatory and antifungal properties to treat fungal keratitis (Yu et al. 2022). Furthermore, germination — the transformation from conidia to invasive hyphae — is considered the most crucial stage in *Aspergillus* pathogenesis. Honokiol may act as an antifungal agent by preventing in vitro biofilm development and adherence. Dectin-1-dependent immune recognition, induced by *A. fumigatus*, can reduce fungal survival through activation of Clec7a (Zhan et al. 2023). The novelty of this research highlights targeting the Dectin-1/p38 MAPK pathway, a crucial mediator in inflammatory responses. Our objectives include evaluating the efficacy of NTH@Pn in inhibiting this pathway and assessing its impact on the corneal epithelium and underlying fungal layers. Our study introduces a novel therapeutic strategy for FK management, aiming to target fungal pathogens while mitigating inflammation for improved treatment outcomes and preservation of ocular health.

**Table 1:** Types of fungi in the application of fungal keratitis

S. No	Type of fungi	Material	Application and function	References
1	*Candida albicans*	Antibiotics	Pre-existing drugs on ocular *Candida* biofilm through in vivo and ex vivo studies	Petrillo et al. (2023)
2	Filamentous fungus	Natamycin	Better visual acuity, with fewer adverse events	Hoffman et al. (2022)
3	*Candida albicans*	Ofloxacin and nepafenac hydrophobic drugs incorporated in zinc ions (Zn^2+^) tagged polyvinyl acetate phthalate	Bioavailability of topically applied drugs to treat fungal eye infection	Chella Daisy et al. (2021)
4	*Aspergillus fumigatus*	Organo-selenium compound	Reducing fungal load and protecting host tissues in fungal keratitis	Yu et al. (2024)
5	*Candida albicans*	Ocular gel and Itraconazole	Advanced treatments in fungal keratitis	Permana et al. (2021)
6	*Aspergillus fumigatus*, *Fusarium solani*	Phytomolecule-coated ZnO nanoparticles	Good cytocompatibility to human corneal epithelial cells and keratinocytes cell lines	Khan et al. (2021)
7	*Aspergillus flavus*	Nanomicelles	Treatment for fungal keratitis	Sathe et al. (2024)
8	*Candida albicans*	β-Sheet forming peptide amphiphiles	Treatment for fungal keratitis	Wu et al. (2015)
9	*Candida albicans*	Amphotericin B	Treating fungal keratitis	Roy et al. (2019)
10	*Fusarium*, *Aspergillus*, and yeast species	Amphotericin B liposomal formulation	Prolong ocular drug delivery and reduce dosing frequency	Mishra et al. (2020)

## Materials and methods

### The synthesis method of nanozyme anionic hydrogel coating (NHC)

#### The CNT nanozyme fabrication process

The following approach was used to produce the CNT nanozyme. Five milligrams per milliliter of carboxyl acid functionalized multiwalled CNTs and 25 mg of itraconazole were dissolved in 100.0 mL of deionized water and agitated at 600 rpm for 5 min to ensure a uniform solution. The mixture was heated at 50 °C for 3 h after the pH was adjusted to 7.4 using a 1.0 M NaOH solution. After that, the above mixture was rinsed with deionized water thrice and centrifuged at 6000 rpm for 10 min. The final product was desiccated at 60 °C overnight to prepare it for future usage and then kept at 4 °C.

#### Preparation of hydrogel precursors

HA was prepared following an established protocol (Shi et al. 2024). To summarize, the HA solution (5.0 mg/mL, 100.0 mL) was gradually supplemented with the following chemicals: ethylenediamine dihydrochloride (3.8 g), NHS (6.5 g), EDC (10.6 g), and 4-dimethylaminopyridine (4.0 mg). The mixture was stirred at 25 °C for 1 day after adjusting the pH to 5.0. Dialysis tubing having a cutoff range of 8000–14,000 Da for 2 days was used to eliminate any unreacted components or by-products after the pH was further lowered to 7.0. Then, the white foam HA is obtained by lyophilizing the dialysis tubing with the mixture for 48 h. After that, the above mixture was added dropwise to 250 mL of TFB ethanol and centrifuged at 5000 rpm for 10 min after letting the mixture stand at an ambient level of heat for a week. The final product was washed thrice with ethanol and underwent 48 h of freeze-drying under vacuum (Garc et al. 2024).

#### Synthesis of NHC

**Scheme 1:**
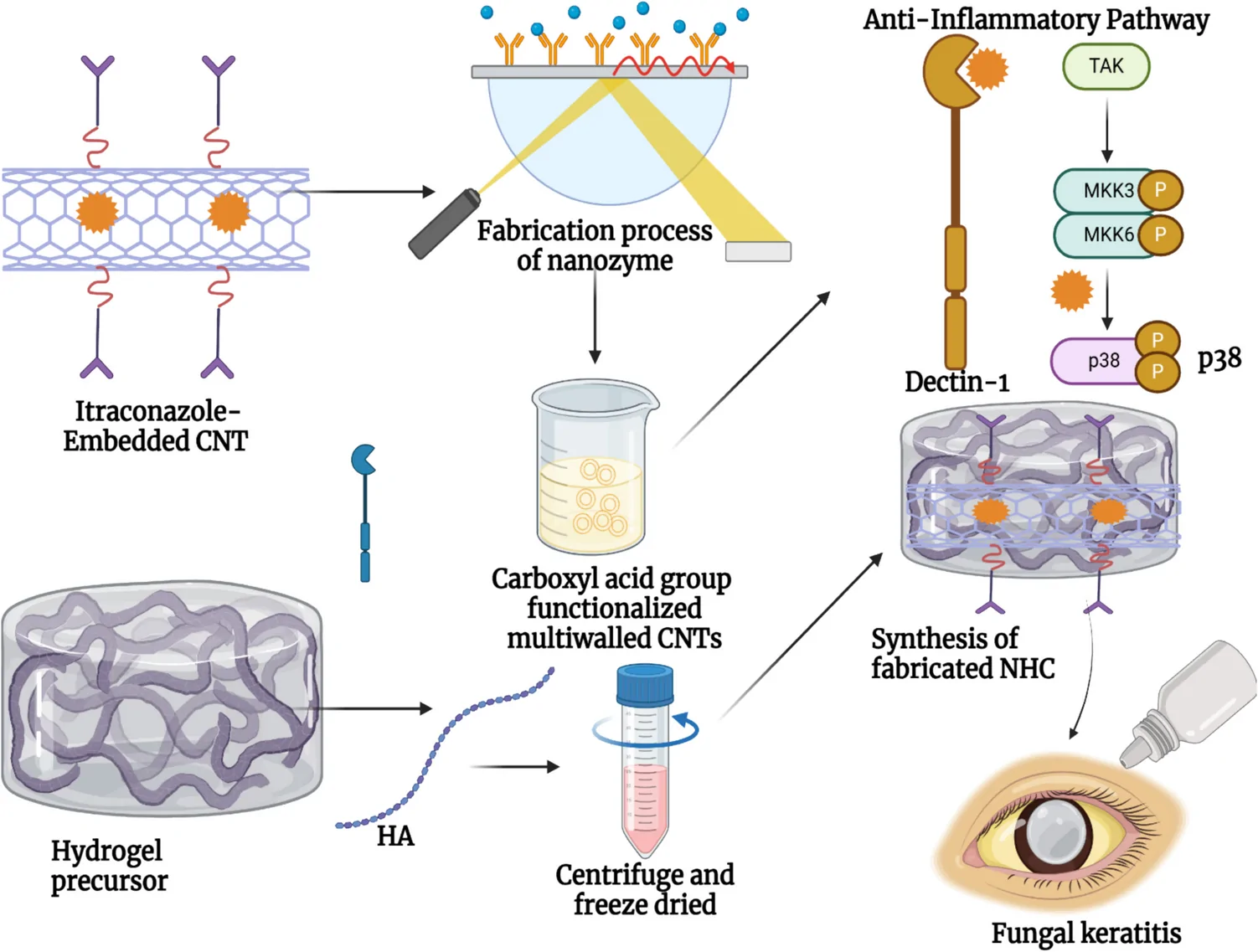
Preparation and application of NTH@CNT/IZ

The Schiff base condensation processes were used to prepare NHC. For synthesis, 400.0 mg of polyurethane (PU) and 250.0 mg of itraconazole were dissolved in 100 mL of PBS and then.

ultrasonicated at 65 °C for 30 min. Then, 1500.0 mg of HA and 4.0 mg of CNT were dispersed in 100.0 mL of PBS, and oscillating the mixture caused the CNT to disperse completely. Moreover, blend the above solution volume for volume for 5 min, and keep it at room temperature. The final product was termed NHC. The preparation process of the synthesized composite NTH@CNT/IZ is illustrated in scheme 1.

#### Characterization of NTH@CNT/IZ

The molecular composition and structure of the CNT@NHC nanozyme were examined using a transmission electron microscope (TEM) fitted with an NEC JEM-1200EX (Japan). To investigate the NHC morphologies, a scanning electron microscope (SEM Zeiss Gemini SEM 360 (Germany)) and a commercial system (VG Microtech ESCA3000) with Mg Kα and Al Kα radiation sources to conduct X-ray photoelectron spectroscopy (XPS) experiments at a base pressure of 3 × 10^−10^ were utilized. A hemispherical energy analyzer with an overall energy resolution of about 0.8 eV obtained the spectra at a normal takeoff angle of 45°. At 285.0 eV, the C1 s binding energy was used as a reference for the binding energy scales (Okpalugo et al. 2005). The readings were acquired using a Swiss-made SevenCompact S210 equipment. For this purpose, NHC rheological characteristics were measured using a TA Instruments (UK) AR-G2 rheometer. For this zeta potential recording, the Leici Instrument (based in Shanghai, China) manufactured a portable dissolved oxygen meter to ascertain the water oxygen concentration. The ability of 3,3′,5,5′-tetramethylbenzidine (TMB) to imitate several enzymes is due to its oxidation when hydrogen peroxide is present. To summarize, a combination of a CNT solution (20 µg/mL, 50.0 µL), H_2_O_2_ (500 mM, 200.0 µL), and TMB (16 mM, 100.0 µL) was heated, while variables such as pH, CNT concentration, H_2_O_2_ concentration, TMB concentration, and reaction temperature were varied. For signals with a wavelength range of 550 to 750 nm, employ a NanoDrop™ One. In the presence of H_2_O_2_, ESR was able to identify OH alterations in CNT (Shi et al. 2024).

#### In vitro antifungal activity and CFU

The agar well diffusion method was used to investigate the antifungal activity of NTH@CNT/IZ on *A. fumigatus* derived from ATCC® 204,305, China. The fungi were evenly spread onto Luria–Bertani (LB) agar plates using sterile cotton swabs after an overnight culture (100 µL). A micropipette was used to make 10-mm diameter wells on LB plates. The wells were then filled with itraconazole (as a positive control), CNT, NHC@CNT, and NHC@CNT/IZ samples at a concentration of 50 µg/mL. These filled plates were incubated (in triplicate) at 37 °C for 24 h. Millimeters (mm) were the unit of measurement for the inhibitory zone. The fungus method of counting colonies was used to find an effective antifungal assessment. In brief, the incubation of fungal solutions started with a concentration of 1 × 10^4^ CFU mL^−1^. The next step was to spread 100 mL of the diluted fungal cells onto areas of solid LB agar. After incubating the plates at 37 °C for one night, the cultures were counted to determine the viability loss induced by the control, IZ, CNT, NHC@CNT, and NHC@CNT/IZ samples. The exposed outcomes were obtained from three rigorous self-determining experiments.

#### In vitro anti-biofilm activity against fungi

The NTH@CNT/IZ was tested against *A. fumigatus* to see whether it had any antifungal effects. The culture was given the green light to flourish on sterile 1 × 1 cm glass cubes set in 24-well plates that contained 1.5 mL of potato dextrose broth containing 50 µg/mL NTH@CNT/IZ. The plates were incubated at 37 °C for 48 h. The liquid was drained from the resulting growth phase, and the glass fragments were collected. They were then stained with 0.4% crystal violet and examined at a × 40 magnification (Nikon inverted light microscope (Nikon, Japan)).

#### Cell culture

ARPE-19 cell lines sourced from the Shanghai, China-based Cell Bank of the Chinese Academy of Sciences. The cells were cultivated in DMEM, a medium containing a lot of glucose, including 10% FBS and 4.5 g/L glucose. The cultures were maintained at 37 °C in a humidified room with 5% CO_2_.

#### Cell viability assay (MTT)

The cell lines ARPE-19 were planted into every well of a 96-well culture plate. Two hours prior to treatment, participants in all studies received different dosages of CNT. To cause harm to the cells, they left the cultivated cells exposed to 5 µM CNT for a full day. After aspirating the culture media, the MTT assay involved adding a 5 mg/mL MTT solution in PBS and incubating it for 1.5 h to produce formazan. The next step was to dissolve the formazan crystals in 1 mL of DMSO and then to measure the readout at 570 nm and the background absorbance at 620 nm (Stat FAX 303 plate reader).

#### Evaluation of ROS

ROS test kit (50101ES01, YEASEN, China) was used to evaluate the levels of ROS. The cells treated with tested samples and culture media before were resuspended, and then a DCFH-DA solution was added. The incubation period for the cells was between half an hour and 4 h at 37 °C, followed by a 3–4 min centrifugation run at 4 °C using 600 × rpm. Resuspending the solid particles in the correct volume of phosphate-buffered saline (PBS) allowed us to measure ROS levels after removing the liquid supernatant and two rinses with PBS.

#### Assessment of nuclei using DAPI analysis

For fluorescence microscopy examination of nuclear structure and apoptotic fragments, DAPI and cell-penetrating nucleic acid stains are appropriate. In 6-well culture plates, ARPE-19 cells were cultivated with the prescribed dosage of CNT while keeping a constant cell density of 2 × 10^1^ cells/well in order to stain the nuclei. The cell nuclei were observed using fluorescent microscopy at a × 10 magnification lens (Olympus Optical, Tokyo, Japan) after a 10 min of incubation under room temperature indistinct light and a PBS rinse.

#### Cell apoptosis through Dectin-1/p38 pathway

Propidium iodide labeling of DNA fragments allowed for the identification of apoptotic cells by flow cytometry. Reportedly, a sub-G1 peak in a hypotonic phosphate-citrate buffer with a quantitative DNA-binding dye, such as PI, suggests that the DNA has fragmented. The histogram to the left of the G1 peak shows that apoptotic cells, which have lost their DNA, will show less staining. The initial step in cultivating ARPE-19 cell lines was to use a 24-well culture plate. After being treated with 100 µg/mL for 2 h, the cells were left to incubate with CNT for 24 h to induce cell damage. The floating and adhering cells were treated overnight at 4 °C in the dark with 750 µL of a hypotonic buffer that included 50 µg/mL PI in 0.1% sodium citrate with 0.1% Triton X-100. The subsequent step was to run the samples via a Becton Dickinson FACS flow cytometer. The FACS method yielded 104 occurrences (Qiu et al. 2018).

#### Real-time PCR

Three, one, and five days post-injection, researchers collected mice corneas (*n* = 5/group/time) for real-time PCR (RT-PCR). At the same time, the total RNA was recovered from C57BL/6 mice corneas.

### Mice models infected with *A. fumigatus*

#### Grouping and treatment

All animal procedures were approved by the Institutional Animal Care and Use Committee at Affiliated Hospital of Jiangnan University, China. Five groups of mice developed keratitis after contracting an *A. fumigatus* infection. There are six animals in each group. There are five groups: one is the control, two is the IZ (positive control), three is the CNT, four is the NTH@CNT, and five is the NTH@CNT/IZ. The 8% chloral hydrate was employed on the mice to induce the effect, and a septic microliter syringe (10 µL; Hamilton Corp., Bonaduz, GR, Switzerland) was utilized to administer 1.5 µL of *A. fumigatus* conidia suspension (1 × 10^8^ CFU/mL) into the neural tube of the mice. The model was induced with a channel and injecting conidia into the stromal layer of the right cornea. A random selection process was used to choose infected mice for both the control and sample treatment groups. The procedure for the control group involved dripping 4 µL of PBS into the right eye three times daily. In a similar vein, the CNT treatment group received 1.5 mg/mL of IZ, CNT, NTH@CNT, and NTH@CNT/IZ. After 1, 3, and 5 days of infection, the degree of keratitis was assessed. These results were revealed using histopathological investigations (Liu et al. 2024).

#### Histopathological evaluation

For histopathological studies, the eyes of infected mice 3 days after infection in both the CNT and PBS groups were removed and examined. Cut the eyeballs into histologic sections that were 5 µm thick after embedding them in paraffin, following xylene deparaffinization, hematoxylin and eosin staining, and ethanol hydration according to a concentration gradient. After encapsulation, an original magnification × 40 microscope was used to image corneal slices.

#### Statistical analysis

All experiments were done three times. Mean ± SEM or median was the way the data were displayed. The Mann–Whitney *U* test was used to evaluate any differences in clinical scores between the two groups. One-way ANOVA and Bonferroni’s post hoc test were used to compare the means of the groups. *p* < 0.05 was used to define significance.

## Results

**Fig. 1:**
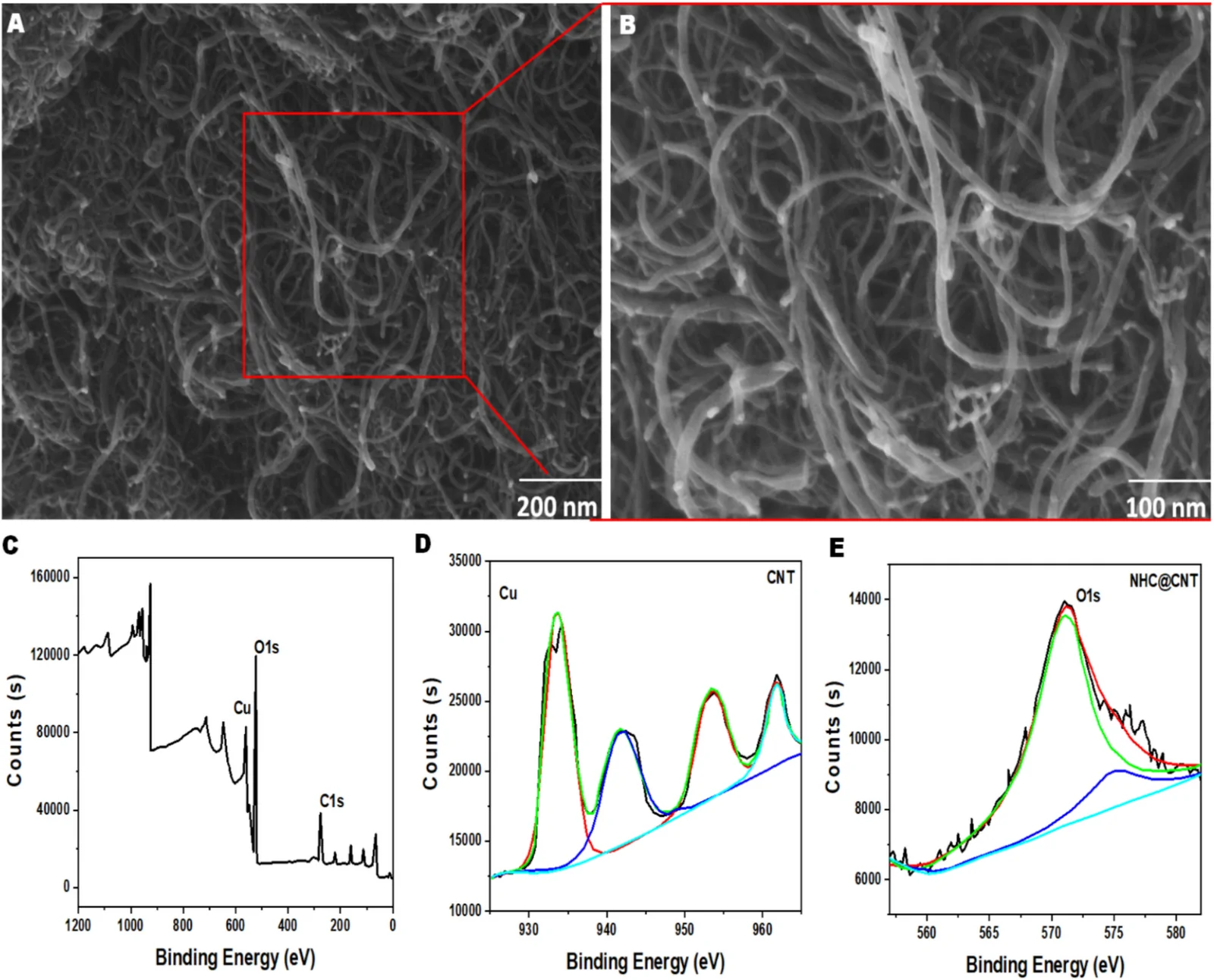
Physical characterization of CNT and NHC. SEM images of NHC@CNT (**A**, **B**). XPS spectra of tested samples (**C**). Binding energy with XPS of CNT and NHC@CNT (**D**, **E**)

**Fig. 2:**
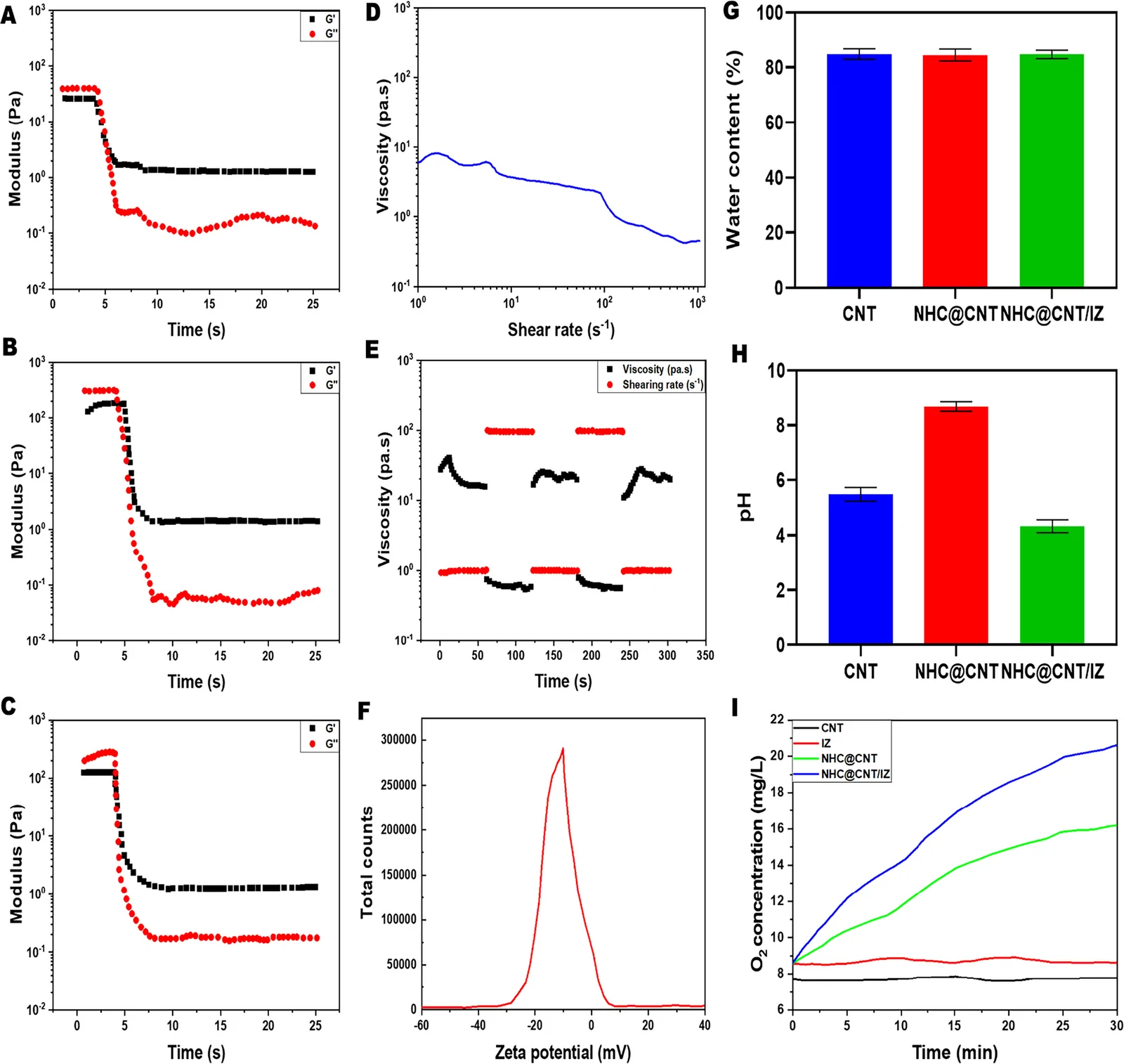
Characterization of CNT and NHC. The fluctuations in storage moduli G′ and loss moduli G″ (**A**–**C**). Viscosity-shear rate curves (**D**). Thixotropy test (**E**). Zeta potential of NHC (**F**). Water content (**G**). pH of tested samples (**H**). O_2_ generating performance of tested samples with different time intervals (**I**)

**Fig. 3:**
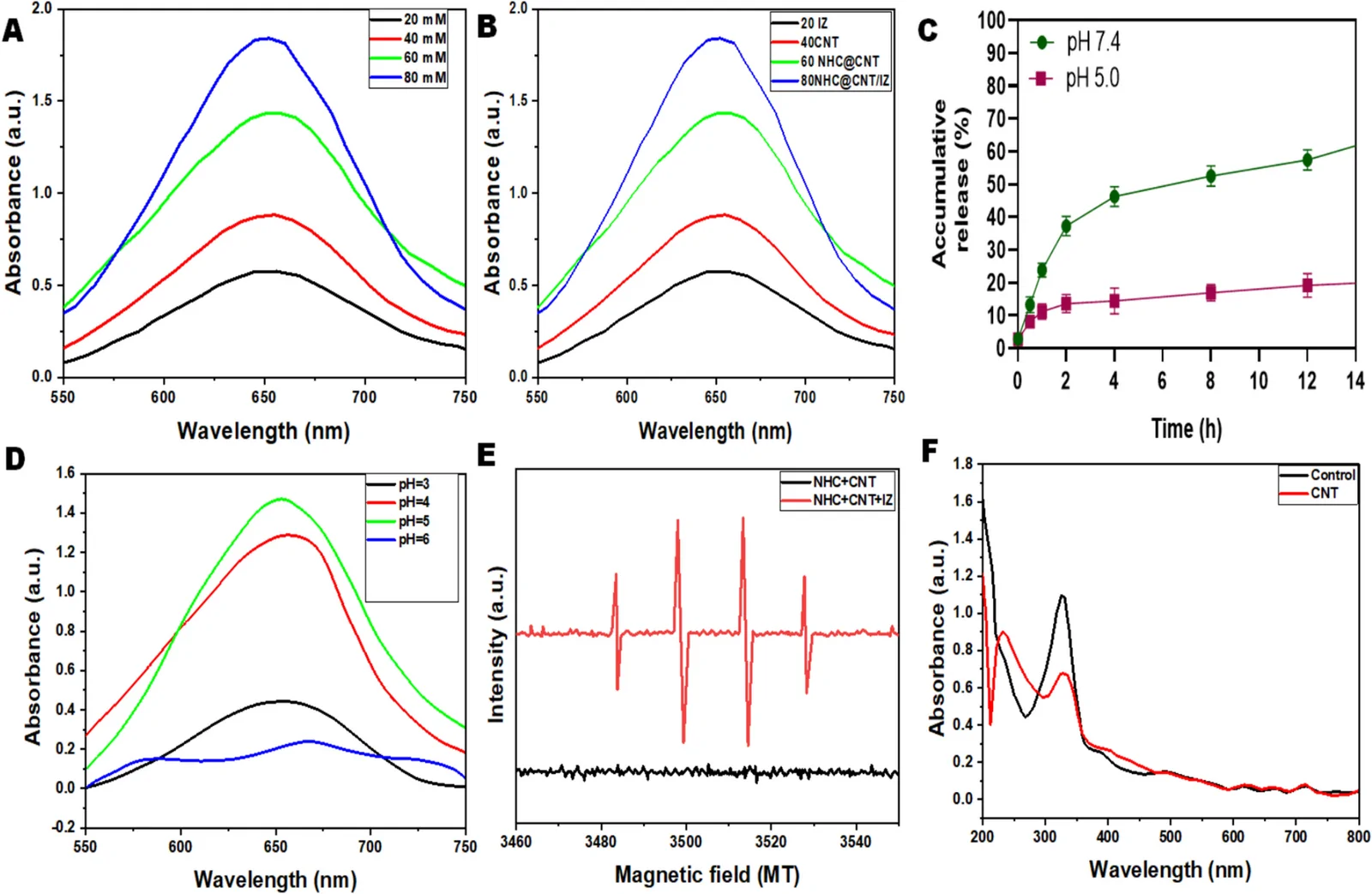
Evaluation of enzyme mimic activity. The absorption spectra of the reaction systems with different compositions with different materials (**A**, **B**). The accumulative drug release at different pH of NHC@CNT (**C**). Absorption spectra of prepared material with different pH (**D**). Magnetic field of the NHC@CNT (**E**). Absorption spectra of autoxidation with CNT (**F**). *p < 0.01 statistical significance was observed

**Fig. 4:**
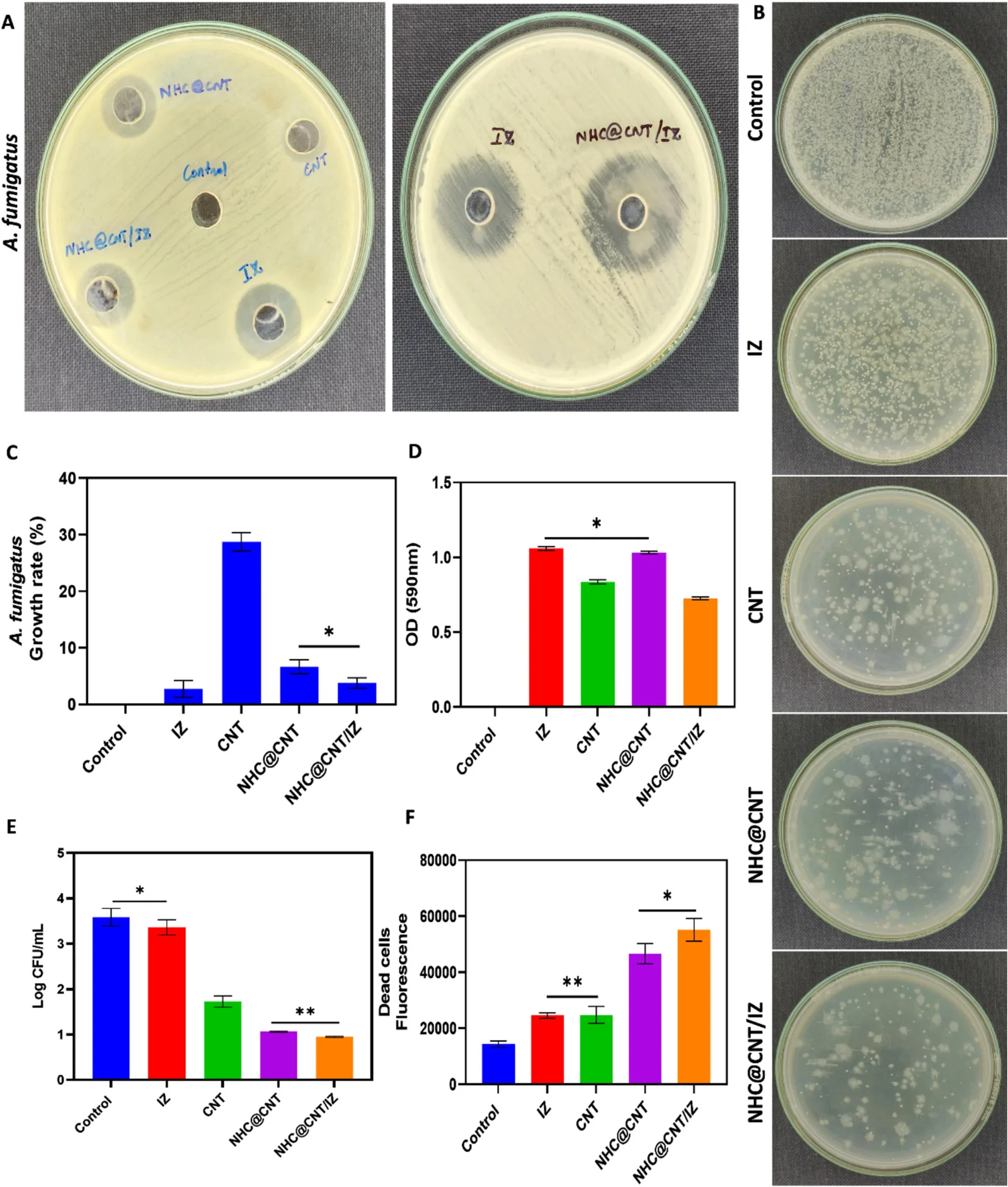
Antifungal activity on the zone of inhibition on *A. fumigatus* (**A**). CFU counting done with different types of sample materials (**B**). Growth of inhibition % (**C**). OD value absorbance at 590 nm (**D**). Fungal viability CFU counting with log CFU/mL (**E**). Fluorescence of dead fungal cells expressed at different materials (**F**). *p < 0.01 significant difference between control

**Fig. 5:**
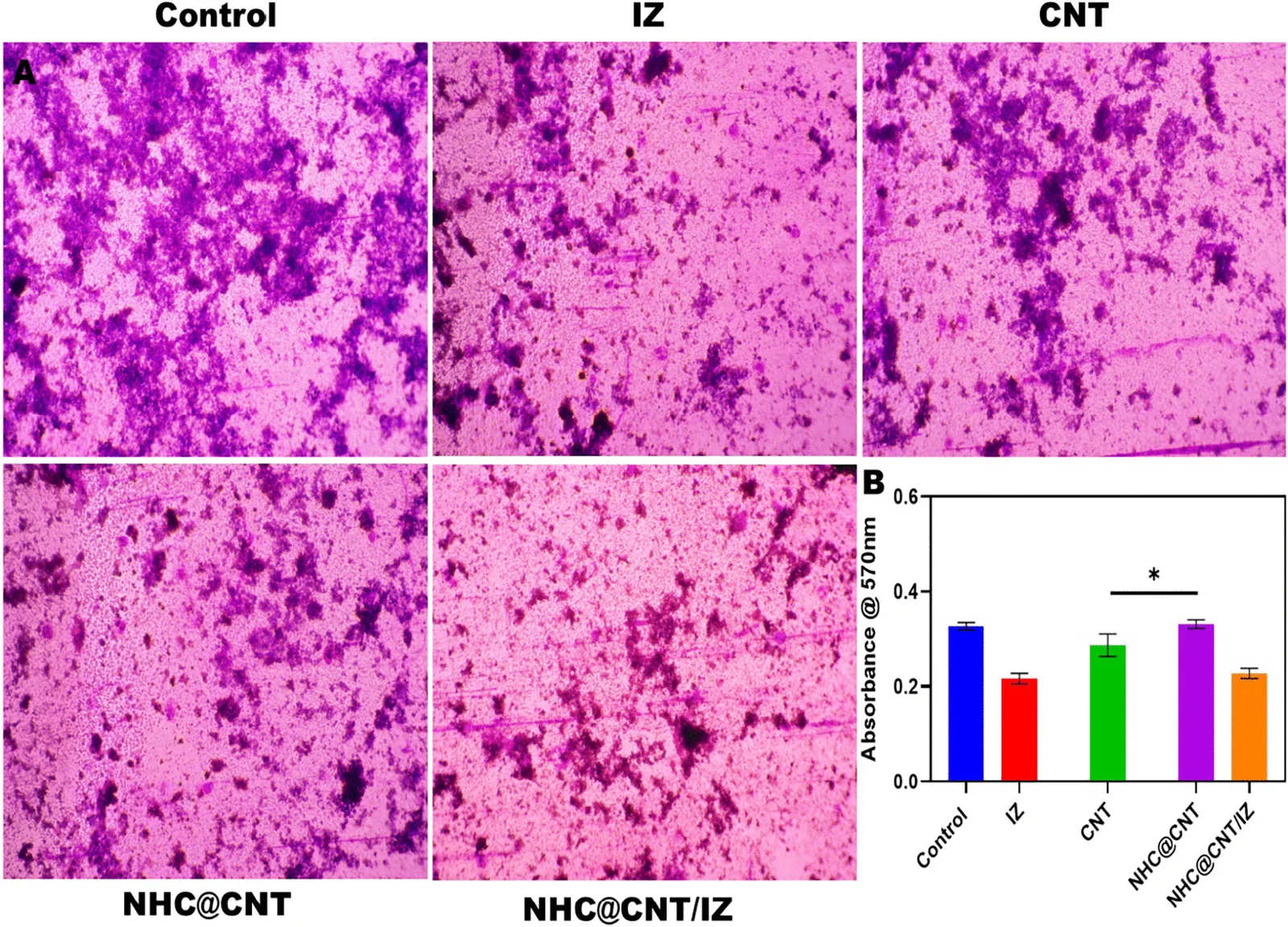
Anti-biofilm activity done through *A. fumigatus* at different samples at 100 µm with × 10 magnification (**A**). Absorbance of anti-biofilm activity with 570 nm (**B**). Statistical differences among groups were observed

**Fig. 6:**
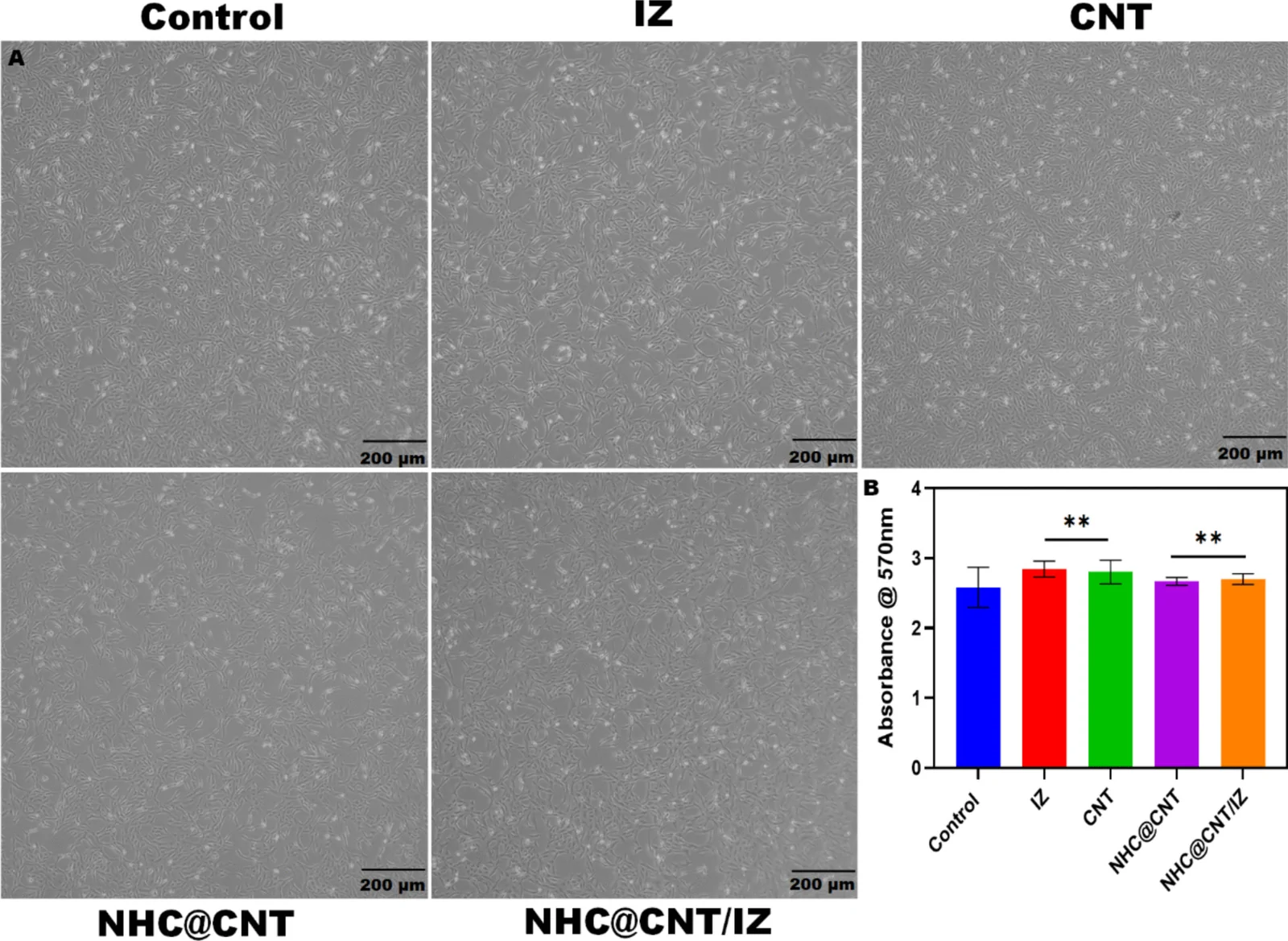
Cell viability of ARPE-19 cell line morphological observation done through phase contrast microscope treated with different samples (**A**). Absorbance through 570 nm (**B**)

**Fig. 7:**
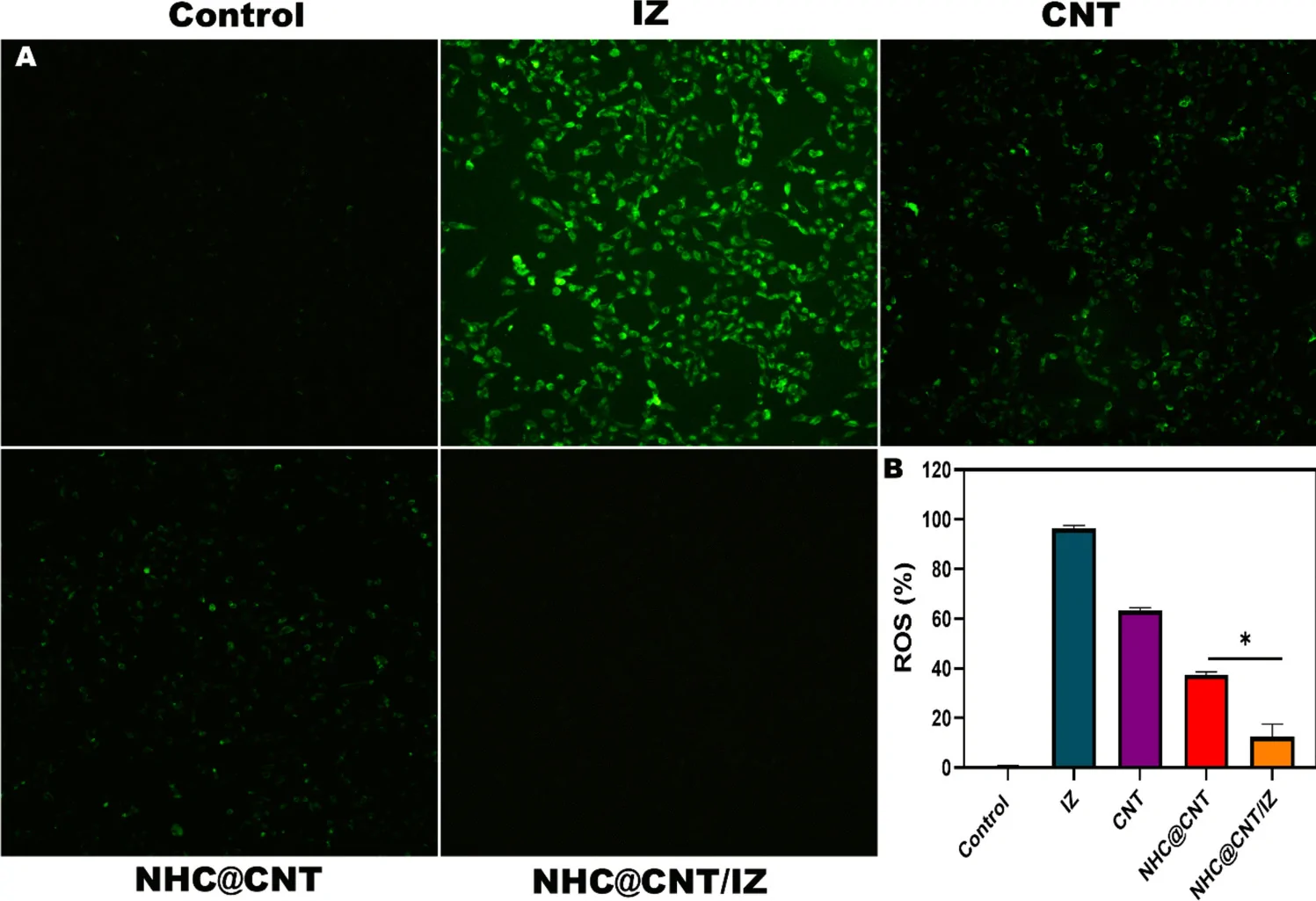
ARPE-19 cell lines were subjected to oxidative stress when exposed to different samples. ROS production in ARPE-19 cells following a 24-h exposure to maximum 100 µg/mL, 100 µm, and × 10 magnification (**A**). ROS percentage with significant asterisk exhibits statistical significance among groups, **p < 0.001 (**B**)

**Fig. 8:**
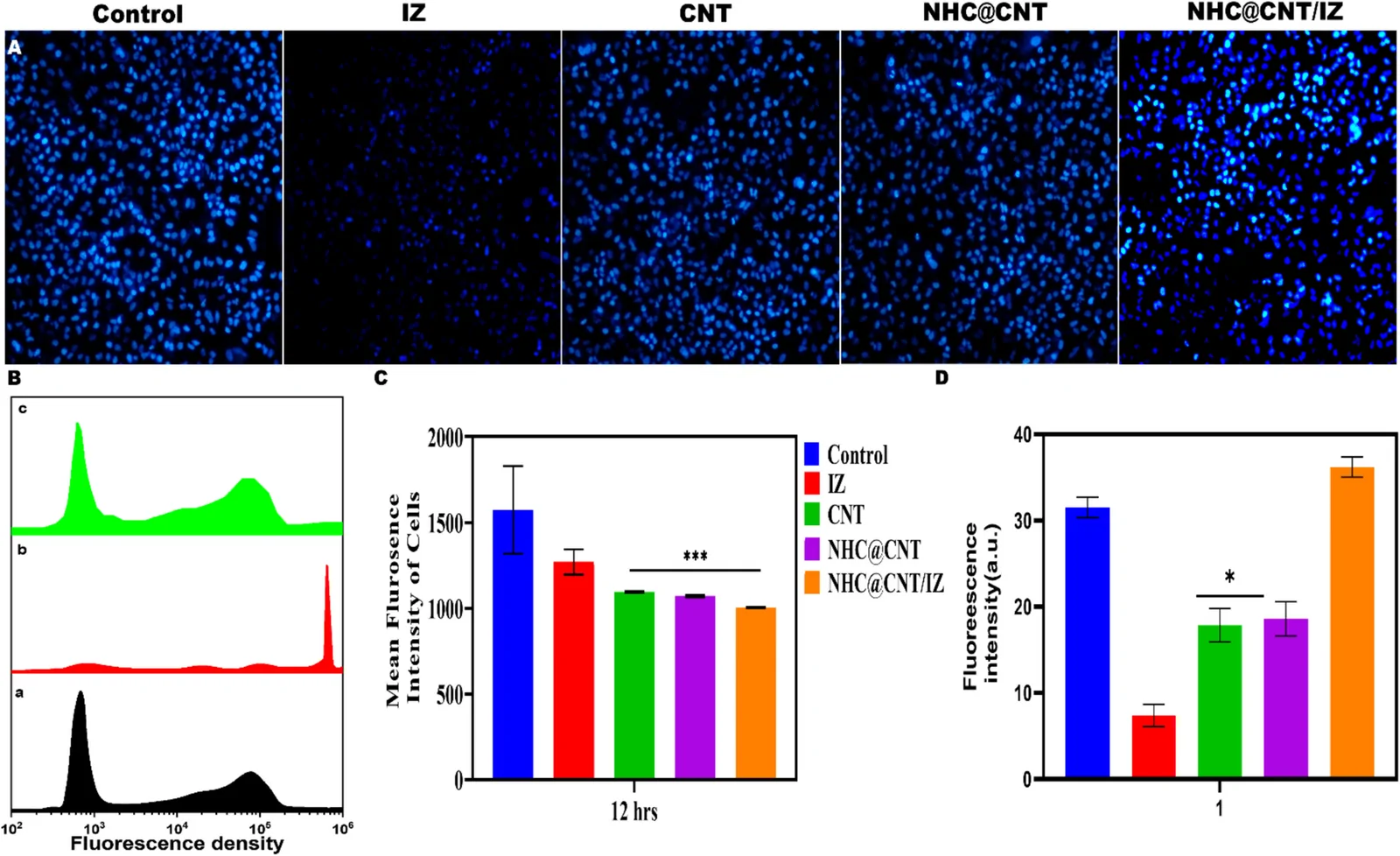
Blue fluorescent dye (DAPI-negative) was used to label the nuclear DNA of proliferating cells. The presence of high levels of blue fluorescence of chromatin condensation at 100 µm and the magnification of × 10 (**A**). Fluorescence density of ARPE-19 cells measured by flow cytometry (**B**). Cell proliferation for ARPE-19 cell lines with various periods of 12 h was examined with significant variations, ***p < 0.0001 (**C**). Fluorescent intensity was measured with sample (**D**)

**Fig. 9:**
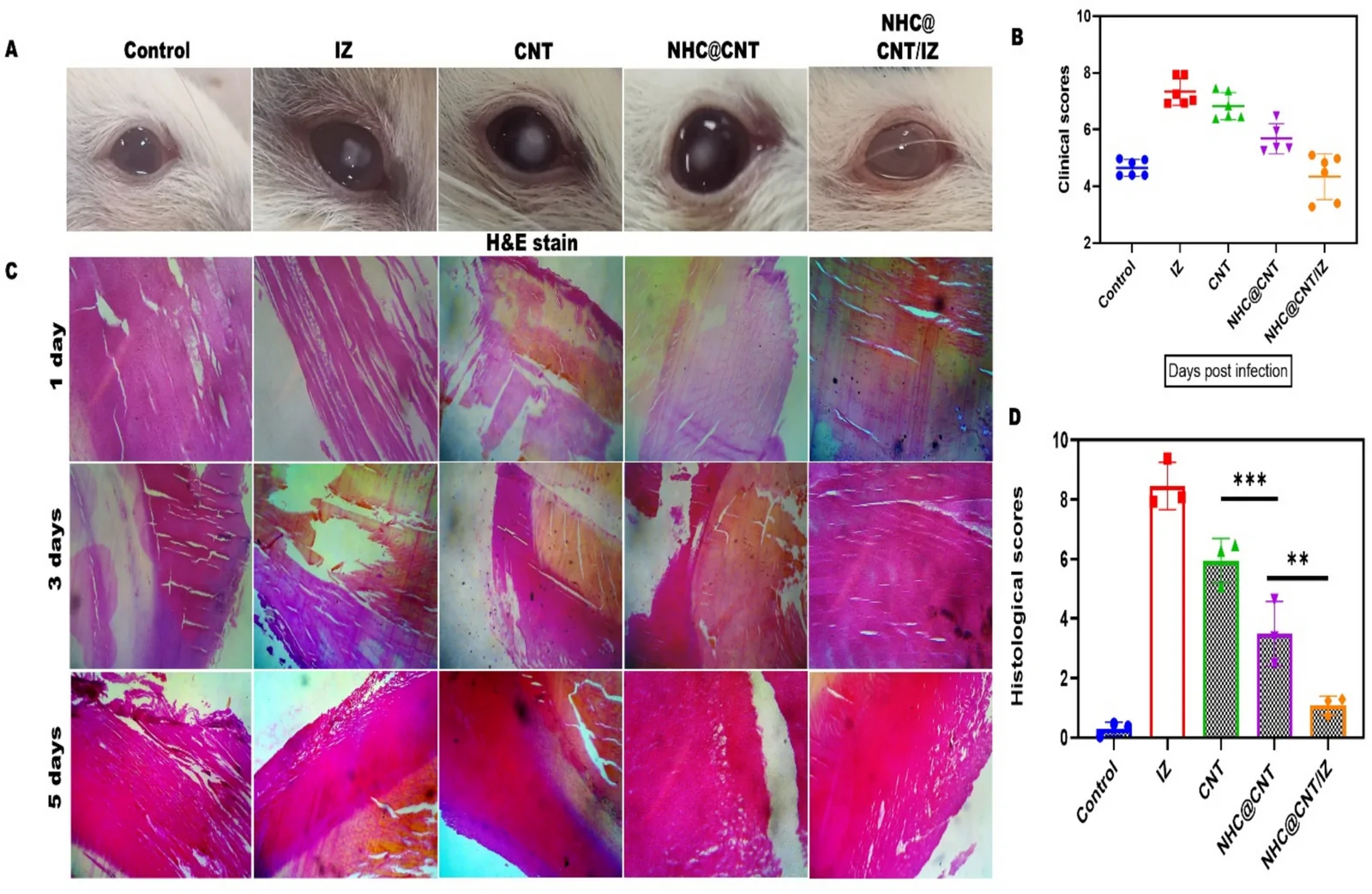
The severity of fungal keratitis infection (**A**). Clinical scores of before and after treatment (**B**). HE staining of the cornea in normal and treated groups corneal infection with *A. fumigatus* in mice after treatment with 1, 3, and 5 days (**C**). Histological scores were observed through statistical analysis (**D**). The asterisk indicates statistical significance among groups: **p < 0.001, ***p < 0.0001

CNT nanocomposite hydrogels exhibit a certain level of complexity and sensitivity. The current investigation observed that the hydrogels exhibited a morphological structure characterized by physical interactions with CNTs (Fig. 1A, B). The morphology of NTH@CNT was observed as a tube-like structure. Moreover, the chemical composition of prepared carbon nanotubes (CNT) and nitrogen-doped holey graphene (NHC) was examined using X-ray photoelectron spectroscopy (XPS) analysis. The XPS showed a spectrum of the functionalized CNT, and NHC@CNT displayed two peaks associated with carbon (C1 s) and oxygen (O1 s) as shown in Fig. 1C. The graphitic sp2 structure of the multiwalled carbon nanotube (MWCNT) framework is 935.3 eV. Besides, the peaks at 571 eV are designated for carbonyl (–CQO) and carboxylic HO–CQO functionalities in NHC@CNT–COOH. The binding energy of carbon nanotubes (CNT) and nitrogen heterocyclic compounds (NHC) was attached to CNT (Fig. 1C–E). The hydrogel SEM images exhibit a hexagonal pattern within a frozen layer of water. Over time, additional holes begin to develop at the corners of the hexagonal shape. Subsequent exposure to the chamber conditions resulted in the emergence of additional cavities on the observed surface, along with a cellular-like structure (Fig. S1). Here, the variations of storage moduli G′ and loss moduli G″ are characterized in CNTs and NHCs (Fig. 2A–C). The viscosity-shear rate curves, thixotropy assessment, zeta potential of NHC, water content, pH, and O_2_ generating performance of tested samples were examined with different time intervals as depicted in Fig. 2D–I. Because of their photothermal action, CNTs play a pivotal role in regulating drug delivery in the CNTs/hydrogel drug delivery system. By turning on photo-irradiation, they can speed up the release of the CNT reported. The activity of enzyme mimics has been evaluated in the present study. The absorption spectra for a range of materials, pH levels, and compositions were reported with the reaction system (Fig. 3A–D). H_2_O_2_ concentrations and autoxidation absorption spectra were measured using CNT (Fig. 3E, F). The agar well diffusion findings demonstrated that at 50 µg/mL, NTH@CNT/IZ successfully inhibited *A. fumigatus*. With higher doses of NTH@CNT/IZ, the inhibition zone expanded. Comparing NTH@CNT/IZ against CNT and NHC@CNT, the latter two showed the narrowest inhibition zones and percentage of inhibition growth. Some sample materials are used for CFU counting while others are not. The absorbance at 590 nm is the OD value. Counting of fungal CFUs using log CFU/mL is available as an expression of fluorescence by dead fungal cells on various substrates (Fig. 4A–F). Like the same, this study found that treatment with 50 µg/mL NTH@CNT/IZ significantly reduced biofilm formation of *A. fumigatus* compared to the control group. The COMSTAT study showed that control glass pieces with a thickness of around 5 µm had a dense formation of *A. fumigatus*, as shown in microscopic photos. In contrast to the results seen with the commercial medicine itraconazole, further treatment with NTH@CNT/IZ (50 µg/mL) against *A. fumigatus* led to weaker adhesion of biofilm development. Itraconazole served as the positive control in all trials (Fig. 5). Assessment of ARPE-19 cell survival and functionality revealed no morphological alterations when the samples were treated with carbon nanotubes, as observed through a phase contrast microscope during morphological observation. There were no morphological changes observed when treated with CNT, NHC@CNT, and NHC@CNT/IZ (Fig. 6). ARPE-19 cell lines were exposed to several substances, resulting in oxidative stress in our investigation. Reactive oxygen species (ROS) generation was measured in ARPE-19 cells after a 24-h exposure to 100 µg/mL of the test compound. The percentage of ROS with a significant asterisk demonstrates statistical significance among the group. ROS generation has decreased when treated with NHC@CNT/IZ compared to CNT and NHC@CNT, whereas in the induced group, IZ has increased (Fig. 7). The nuclear DNA of proliferating cells was labeled using a blue fluorescent dye that does not react with DAPI. The observation of elevated levels of blue fluorescence indicates the condensation of chromatin. Cell proliferation was assessed in ARPE-19 cell lines at different time intervals of 12 h. The measurement of fluorescent intensity was conducted using the samples CNT, NHC@CNT, and NHC@CNT/IZ, which has increased the proliferation percentage of cells (Fig. 8A–D). In this study, the apoptosis rate was determined using flow cytometry. Exposing ARPE-19 cell lines to higher concentrations of the samples does not result in cell mortality. The percentage of apoptotic cells decreased to 15–20%, while the percentage of live cells increased. In contrast, the induced model, treated with IZ, had a survival rate of less than 3%. The percentage of apoptosis was examined using statistical methods. The administration of MPO and this particular assay demonstrated a notable decrease in the presence of neutrophils following the therapy (Fig. S2). The impact of various materials’ influence on Dectin-1 and Toll-like receptor 4 synthesis in C57BL/6 corneas when exposed to *A. fumigatus* was examined. The use of different materials effectively suppressed the increase in relative gene expression of Nrf2, IL-1β, TNF-α, IL-8, and IL-6 induced by *A. fumigatus*, compared to the control group (Fig. S3). Previous studies have also demonstrated that OMT decreased IL-1β-induced inflammation and extracellular matrix degradation in osteoarthritis models. It was achieved by increasing Nrf2 and decreasing the NF-κB signaling pathway. This study examined the relative mRNA expression levels of Nrf2, iNOS, and TNF-α. Expression levels of Dectin-1 and GAPDH in the examined samples control, IZ, CNT, NHC@CNT, and NHC@CNT/IZ were all expressed. All experiments were conducted in triplicate, and the results show statistical significance between the groups (Fig. S4). Following infection, the group of mice treated with natamycin and the other group of mice treated with schaftoside both showed decreased corneal opacity and lower clinical scores compared to mice with fungal keratitis alone. Corneal tissue from both the normal and treated groups was subjected to HE staining. Mice were treated for 1, 3, and 5 days and subsequently developed a corneal infection caused by *A. fumigatus*. Statistical analysis was used to observe histological scores. The severity of fungal keratitis infection was higher in the induced group and reduced after being treated with NTH@CNT/IZ. The clinical scores before and after treatment showed reduced inflammation. The residence time of the NTH@CNT/IZ on the ocular surface refers to how long this formulation remains on the eye’s surface before tears or other mechanisms clear it. In this context, NTH@CNT/IZ is likely designed to enhance the residence time through its thixotropic properties, meaning that it can form a gel-like structure on the eye, which helps it adhere longer to the ocular surface. Typically, the residence time for standard eye drops can range from about 20 min, depending on the formulation. However, hydrogels like NTH@CNT/IZ can significantly prolong this time due to their mucoadhesive nature and ability to cross the corneal epithelium. This prolonged residence time would allow for better drug absorption and more effective fungal inhibition, leading to improved treatment outcomes (Fig. 9). Our study shows that integrating these components in a thixotropic anionic hydrogel matrix is advantageous. The hydrogel’s shear-thinning and self-healing properties allow sustained and localized drug release at the infection site, improving drug retention and reducing administration frequency. More importantly, the formulation targets the Dectin-1/p38 MAPK pathway to boost antifungal efficacy and reduce inflammation. The antifungal and anti-inflammatory approach differs from CNT- or itraconazole-based systems, which lack immune modulation or extended ocular surface residence time. Thus, this study introduces a multifunctional therapeutic platform for fungal keratitis with improved clinical potential.

## Discussion

The chemically treated XPS C1s spectra of CNTs show a main peak at 572.4 eV, which is attributed to sp^2^ carbon in the graphitic structure and is visible in all Gaussian-deconvoluted spectra. The sp3-hybridized carbon atoms on the surface of the CNT are responsible for the low intensity peaks at higher binding energies (483.5 and 459.2 eV). Results like these highlight the value of XPS as a surface analysis tool for determining the nature of functional groups and identifying structural flaws on CNT surfaces. The XPS C1s spectra of CNTs before and after chemical treatment indicate the introduction of functional groups and structural modifications on the CNT surface (Okpalugo et al. 2005) (Moraes et al. 2011). The critical need for exploratory qualitative research into the solubilization of CNTs in water-based solutions used to synthesize hydrogels (Adewunmi et al. 2016). Similarly, the hydrogel surface, devoid of adsorption, was pristine and smooth. Numerous flocculated impurities were present on the surface of the hydrogel following adsorption (Gao et al. 2022a). The SEM images of the hydrogel's cross-segments show the H-polymer and H-f-MWCNTcomponents. The H-polymer and H-f-MWCNT hydrogel samples were characterized by highly porous morphologies, with mean pore diameters of 112 ± 17.84 and 77 ± 13.09 µm, respectively. Hydrogels with smaller pores after f-MWCNT addition may have resulted from molecular-level chemical and mechanical relationships between the polymer network and the carbon nanofiller (Garc et al. 2024). XPS was used to discover the composition CuCo/NC. In addition to the successful loading of nanoparticles onto the HG surface, the EDS and elemental analysis by SEM reveal a uniform distribution of CuCo/NC on the surface (Song et al. 2024). The functionalized multiwalled carbon nanotubes (f-MWCNT) participating in the supramolecular polymer architecture, in the nanocomposite hydrogel, were able to do so via hydrophobic interactions and hydrogen bond formation mediated by oxygen groups on their surfaces. Composite hydrogel formulations using carboxymethyl cellulose sodium (CMCs) have the potential to be clinically safe. The human epidermis equivalent that was used as epiderm is readily available for purchase, thus able to evaluate dermal toxicity and skin research concerns without resorting to animal testing (Cao et al. 2023). The nitrogen- and sulfur-doped carbon dot (NS/CD) locations were reported through their fluorescence emissive properties under varied light spectral wavelengths between 270 and 400 nm (Pongchaikul et al. 2023). The assessment focused on the multienzyme-like activity of the as-prepared CuPC, which encompasses peroxidase, superoxide dismutase, and catalase actions and peroxidase-like activity by oxidizing TMB with H_2_O_2_ (Shi et al. 2024). The fungus remarkable capacity to withstand and adapt to its surroundings is likely responsible for this trait. It is possible to create optimized enzyme systems for industrial carbohydrate degradation by modifying the adaptable and efficient enzyme system of *Acinetobacter fumigatus*, although pathogenicity prevents direct use in manufacturing (Tong et al. 2024). It has long been believed that fungal keratitis, one of the most acute eye illnesses, is the leading cause of blindness and visual impairment on a global scale. For a long time, people in rural regions have blamed objects that have come into contact with plant or soil debris for the spread of FK (Ahmadikia et al. 2021). In addition to fungal invasion, high inflammation is the main cause of corneal injury (Sha et al. 2021). A comprehensive investigation into the precise antifungal mechanism of OMT since previous antifungal studies lacked specificity. Important connecting steps in FK formation include biofilm formation and adhesion. In order to infect a host, fungi must first adhere to the tissue surface (Niu et al. 2020). As a similar outcome explains, biofilms, which play a significant role in fungal invasion and pathogenicity, also increase the structural stability of fungi, making them more resistant to environmental stresses (Mayer et al. 2013). *A. fumigatus* biofilm total biomass and fungal viability were both dramatically reduced by AMB and CAS at all concentrations, as reported (Fortes et al. 2023). Because itraconazole and caraway essential oil (EO) both suppress ergosterol synthesis in *A. fumigatus*, the current investigation suggests that the two antifungals may work synergistically (Saremi et al. 2024). The co-culture of bacteria and fungi showed the most potent antifungal activity in the ethyl acetate extract. This result is evident since the inhibition zone’s average diameter reaches 25.67 ± 0.59 mm, which is bigger than the inhibition zone in individual cultures (Octarya et al. 2023). Numerous studies have demonstrated that OMT’s powerful anti-inflammatory properties make it useful in treating a wide range of disorders (Dong et al. 2019). Among the most important agents that cause FK, *A. fumigatus* is a crucial player. The germinating and subsequent production of hyphae by latent spores allow them to propagate throughout the cornea once they have colonized the stroma. The fungus contributes to the cornea demise by its invasiveness, poisons, and adherence. When *A. fumigatus* conidia reach the basement membrane of the corneal epithelial cells, they burrow through it, allowing inflammatory cells to infiltrate. The amount of conidia positively correlates with the severity of inflammation in the cornea and the rate of neutrophil chemotaxis (Gao et al. 2022b). In corneas treated with PBS and guanosine 5′-monophosphate disodium salt (GMP)—and even with TOB eye drops—the colony-forming units of *P. aeruginosa* in corneas treated with GMP/TOB gel (GT Gel) were three orders of magnitude lower. Furthermore, compared to the control groups, the corneas treated with GT Gel had lower MPO levels, suggesting that the infected corneas had less neutrophil infiltration following therapy (Cheng et al. 2022). The anti-inflammatory activity of itraconazole may be due, in part, to its phenylpiperazine ring. In inflammatory diseases like mycosis fungoides and LP, itraconazole may be effective due to its immunosuppressive effect (Tsai and Tsai 2019). The findings indicated that the cell viability decreased to approximately 50% of the control when the concentration of H_2_O_2_ reached 800 µM (Liu et al. 2017). The average cell viability of ARPE-19 cells treated with 120 µg/mL TETRA increased by 22.1% (*p* = 0.001) compared to the control wells without treatment. Following a 48-h treatment period at a concentration of 120 µg/mL TETRA, there was a statistically significant 17.7% increase in the average cellular metabolism compared to the control cells treated with the vehicle. The ARPE-19 cultures exhibited comparable results regarding ROS levels. The cells treated with TETRA at a concentration of 120 µg/mL for 48 h exhibited a significant decrease of 32.6% (*p* = 0.031) in ROS levels compared to the untreated samples (Salimiaghdam et al. 2020). An in vitro investigation has demonstrated that a 1-h treatment is enough to induce a 30–40% inhibitory effect on the growth of RPE cells. In this study, higher quantities of MTX did not result in a stronger inhibitory effect on the proliferation of ARPE-19 cells (Schulz et al. 2021). Suppressing PVT1 enhances survival and inhibits the programmed cell death and inflammatory reaction of ARPE-19 cells induced by high glucose (HG). Functionally, PVT1 interacts with miR-1301-3p to increase the expression of KLF7 and, after that, carries its effects in ARPE-19 cells exposed to elevated glucose levels (HG) (Guo et al. 2022). The biocompatibility of methotrexate (MTX) solutions, at concentrations up to 266 µg/mL, was evaluated in fibroblastic (BJ), RPE (ARPE-19), and photoreceptor (661 W) cells. Additionally, the antiproliferative effect of MTX concentrations against ARPE-19 cells was studied, starting at a concentration of 8 µg/mL (Schulz et al. 2021). This study provides an enhanced understanding of the impact of different doses of curcumin on ARPE-19 cells. Specifically, flow cytometry is used to analyze levels of apoptosis. The study discovered that curcumin did not lead to a notable spike in the rate of apoptosis after treatment with the tested concentrations, as compared to the control group (Carozza et al. 2022). Like the same, the results were similar in the ARPE-19 cells treated with B(e)P, which showed an elevated ROS/RNS value when compared to the cultures treated with DMSO. Genistein, resveratrol, and memantine were the most effective inhibitors in reducing ROS/RNS levels, following the same sequence as the inhibitors that reduced caspase-3/7 and caspase-9 activity (Mansoor et al. 2010). On a similar note, Lulli et al. (2024) showed that ARPE-19 cell viability and proliferation were enhanced by hAME treatment. Even though hAME treatment improved cell viability, it did not prevent oxidative stress–induced cell death (Lulli et al. 2024). Also, there was a reduction in corneal opacity, swelling, ulcer area, and other FK lesions after GLD treatment at 1, 3, and 5 days after the incision. Furthermore, GLD treatment reduced MPO units and stopped neutrophil infiltration of the corneal stroma during an *A. fumigatus* infection as reported (Gao et al. 2022b). Additionally, Nrf2 might further suppress the activation of the NLRP3 inflammasome (Zhou et al. 2023). Gene expression studies showed that ARPE-19 cells reacted differently to tetracycline (TETRA) than cells treated with ciprofloxacin (CPFX). Specifically, ARPE-19 cells upregulated the anti-apoptosis gene BCL2, downregulated the growth and differentiation gene TGF-β1, and showed higher levels of the proinflammatory gene IL-1β (Salimiaghdam et al. 2020). NF-κB controls the production of chemicals that promote inflammation, such as TNF-α and iNOS. Treatment with α−1antitrypsin (A1 AT) brought the levels of retinal TNF-α back to normal levels in a diabetic mice model (Potilinski et al. 2020). A key player in controlling its secretion from cells is the p38 signaling pathway. Additional research with genetic intervention methods may be necessary to confirm the veracity of this finding, as kinase inhibitors may not always be precise enough (Chang et al. 2021). NF-κB controls the production of chemicals that promote inflammation, such as TNF-α and iNOS. Previous findings demonstrated that A1 AT treatment brought the levels of retinal TNF-α back to normal levels in a diabetic mice model. The expression of NF-κB, TNF-α, and iNOS on ARPE-19 cells in the presence of high glucose, A1 AT, and results align with our earlier discoveries (Potilinski et al. 2020). The possibility of voltage-dependent potassium channel activation is quite rare. It was recently reported that ARPE-19 cells exhibit depolarizing voltage-induced currents that are both cationic and anionic, and current values at + 70 mV were determined to be consistent with our experimental results. Correspondingly, stimulating macrophage activation and the production of inflammatory cytokines, Dectin-1 produced by macrophages contributes to the pathophysiology of diabetic cardiomyopathy (Yang et al. 2023). Consistent with earlier findings, BI decreased the expression of LOX-1, phosphorylation of p38MAPK, and JNK in C57BL/6 mice with *A. fumigatus* keratitis and *A. fumigatus*–stimulated HCECs. This finding, with the current study’s network pharmacology analysis, revealed that BI controls FK pathogenesis via the CLR signaling pathway. BI inhibits *A. fumigatus* fungal growth and biofilm formation, increases fungal membrane permeability, inhibits *A. fumigatus* virulence-associated genes, and reduces conidia adhesion to HCECs (Yin et al. 2022). By inhibiting the phosphorylation of Dectin-1 and p38 MAPK, resveratrol (RES) was able to exert its anti-inflammatory effects. The utilization of curdlan, which agonizes Dectin-1 specifically, is used to stimulate HCECs in order to determine if RES exerts its anti-inflammatory effects by regulating the Dectin-1/p38 MAPK pathway in FSK. Curdlan successfully activated HCECs and increased levels of IL-1β, IL-6, and p38 MAPK activation. Reversal of these effects was also possible with RES pretreatment. By inhibiting the Dectin-1/p38 MAPK pathway, as shown above, RES may play a pivotal role in anti-inflammatory strategies (Diao et al. 2024). Concurrently, at the 32 µM concentration that does not harm the cornea of living mice, quercetin interfered with the structure and membrane integrity of *A. fumigatus* hyphae and also reduced their adhesion capacity. To protect mice from *A. fumigatus* keratitis, quercetin reduces fungal load, breaks down hyphae structure, and prevents macrophage inside filtration (Montgomery and Fuller 2020). Drug delivery systems like liposomes, hydrogels, and contact lenses can extend residence time by releasing the drug slowly or retaining it in the eye (Luan et al. 2023). Contrarily, on days 3 and 5, after infection, there were no discernible variations in corneal opacity between the groups treated with natamycin and those treated with schaftoside (Lu et al. 2024). When comparing RA and DMSO treatments, HE staining revealed that RA reduced the number of conidia attached to cells. After 24 h of treatment with RA (250 and 500 µM), the number of PI-colored hypha increased compared to DMSO treatment, according to the results of the PI stain. Conferring to these findings, RA decreased the amount of germinating *A. fumigatus* spores and increased the number of dying hyphae (Wang et al. 2024). When administered to C57BL/6 mice, kaempferol (KAE) improved the outcome of *A. fumigatus* keratitis. In particular, KAE therapy slowed the evolution of *A. fumigatus* keratitis in C57BL/6 mice by reducing clinical scores, inflammatory cell infiltration, and structural corneal damage. These results developed the theory that KAE regulates the inflammatory response to different illnesses. Furthermore, KAE significantly decreased the fungal burden in corneas infected with *A. fumigatus*, indicating that KAE could potentially possess antifungal characteristics (Jia et al. 2022). In conclusion, an important advance in the administration of fungal keratitis (FK) has been made with the prepared nanozyme thixotropic hydrogel anionic coating with itraconazole (NTH@CNT/IZ). Our experimental analyses, including both in vitro and in vivo investigations, have shown an effective and novel treatment strategy. By obstructing the Dectin-1/p38 MAPK pathway, NTH@CNT/IZ coating significantly reduces inflammatory responses in addition to effectively reducing the fungal burden in the cornea. Furthermore, no morphological changes were observed in ARPE-19 cell lines treated with NTH@CNT/IZ, and there was no cell death. Important inflammatory markers that are targeted include TNF-α, IL-1β, and Dectin-1. The encouraging findings of our study highlight the potential of NTH@CNT/IZ as a potent and successful treatment choice for the management of FK. It is necessary to conduct additional research and clinical trials to confirm these results and investigate this strategy in broader applicability to preserve ocular health. NTH@CNT/IZ may represent a therapeutic environment for FK, providing hope for improved treatment and vision preservation for the affected.

## Supplementary information

 Below is the link to the electronic supplementary material.

ESM 1 (PDF 843 KB)

## Data Availability

Data will be made available on request. All the data generated or analyzed during the study are included in this published article.

## Abbreviations

AAT: α-1Antitrypsin
CAS: Caspofungin
CFU: Colony-forming unit
CMCs: Carboxymethyl cellulose sodium
CNT: Carbon nanotube
DAPI: Diamidino phenylindole
DMSO: Dimethyl sulfoxide
EDC: Ethylenediamine dihydrochloride
EO: Essential oil
FDA: Food and Drug Administration
FK: Fungal keratitis
GMP: Guanosine 5′-monophosphate disodium salt
HA: Hyaluronic acid
H_2_O_2_: Hydrogen peroxide
IZ: Itraconazole
KAE: Kaempferol
LB: Luria-Bertani
mm: Millimeters
f-MWCNT: Functionalized multiwalled carbon nanotubes
MTX: Methotrexate
NaOH: Sodium hydroxide
NHC: Anionic hydrogel coating
NS/CDs: Nitrogen- and sulfur-doped carbon dots
OMT: Oxymatrine
PBS: Phosphate-buffered saline
RT-PCR: Real-time polymerase chain reaction
PU: Polyurethane
ROS: Reactive oxygen species
SEM: Scanning electron microscope
TEM: Transmission electron microscope
TMB: Tetramethylbenzidine
XPS: X-ray photoelectron spectroscopy
