# Supplementary material for: Fabrication of nanozyme thixotropic anionic hydrogel for treating fungal keratitis by Dectin-1/p38 pathway
Source: Appl Microbiol Biotechnol. 2025 Jun 26;109:153. doi: 10.1007/s00253-025-13529-8 (PMC12202696; doi:10.1007/s00253-025-13529-8)

Journal name : **Applied Microbiology and Biotechnology**

Manuscript Title : **Fabrication of Nanozyme Thixotropic Anionic Hydrogel for Treating Fungal Keratitis by Dectin-1/p38 Pathway**

Name of author : **Chenchen Zhang*, Jia Li, Xinyue Shen, Jihong Wang**

The affiliation address of the author : **Department of Ophthalmology, Affiliated Hospital of Jiangnan University， Wuxi 214122, China**

***Correspondence to:**

Chenchen Zhang*, NO.1000, Hefeng Road, Wuxi, China.

The e-mail address : Chenchen@12345

Fig S1: SEM images of nanozyme thixotropic anionic hydrogel at different magnifications.

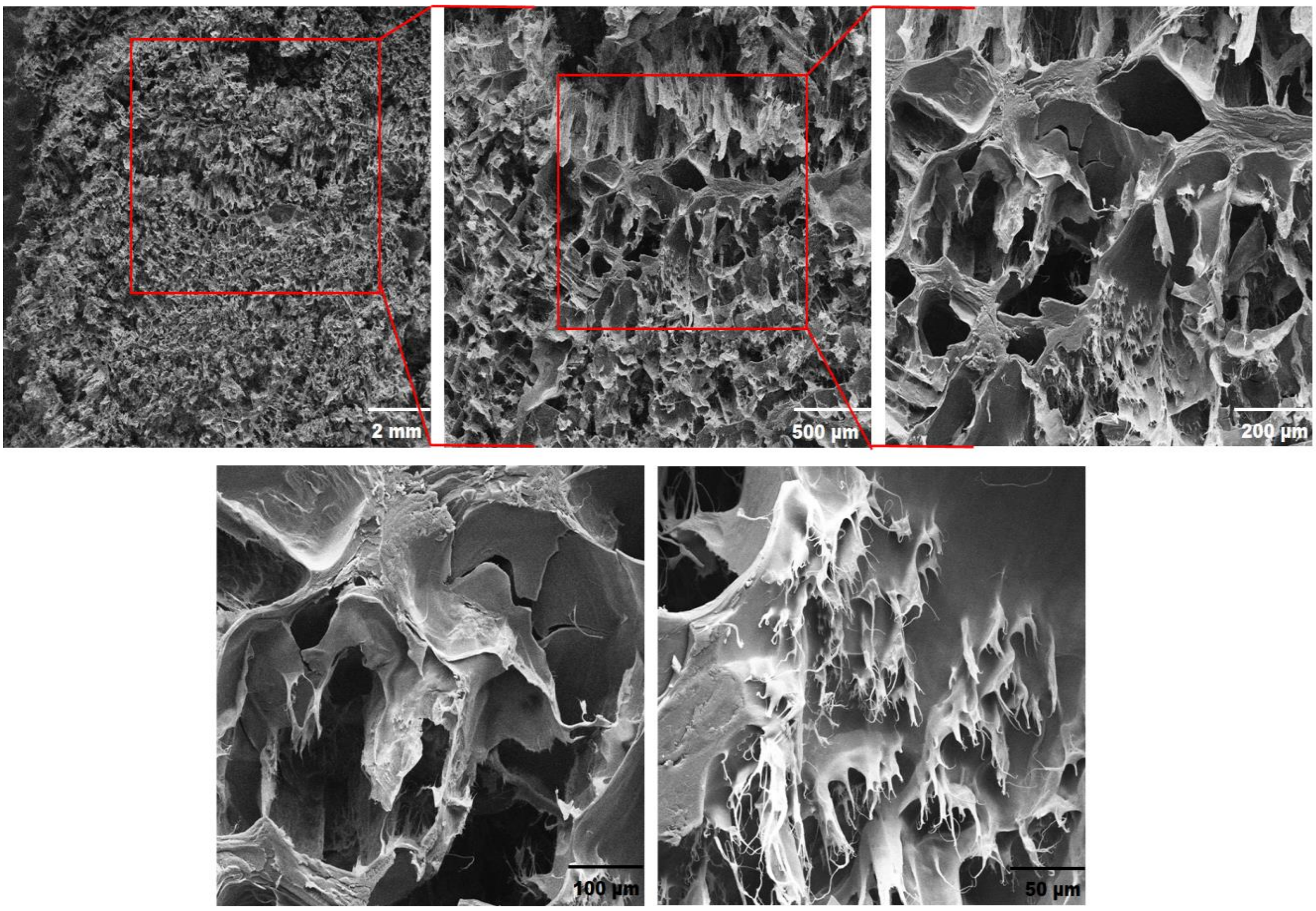

Fig S2: Flow cytometry was used to determine the apoptosis rate. Treatment of ARPE-19 cell lines with higher doses of the samples cause no cell death (A); The apoptosis percentage was analyzed with statistical methods (B); MPO (C); Assay showed significantly reduced neutrophil infiltrate after treatment (D). *p < 0.01 significant differences between materials.

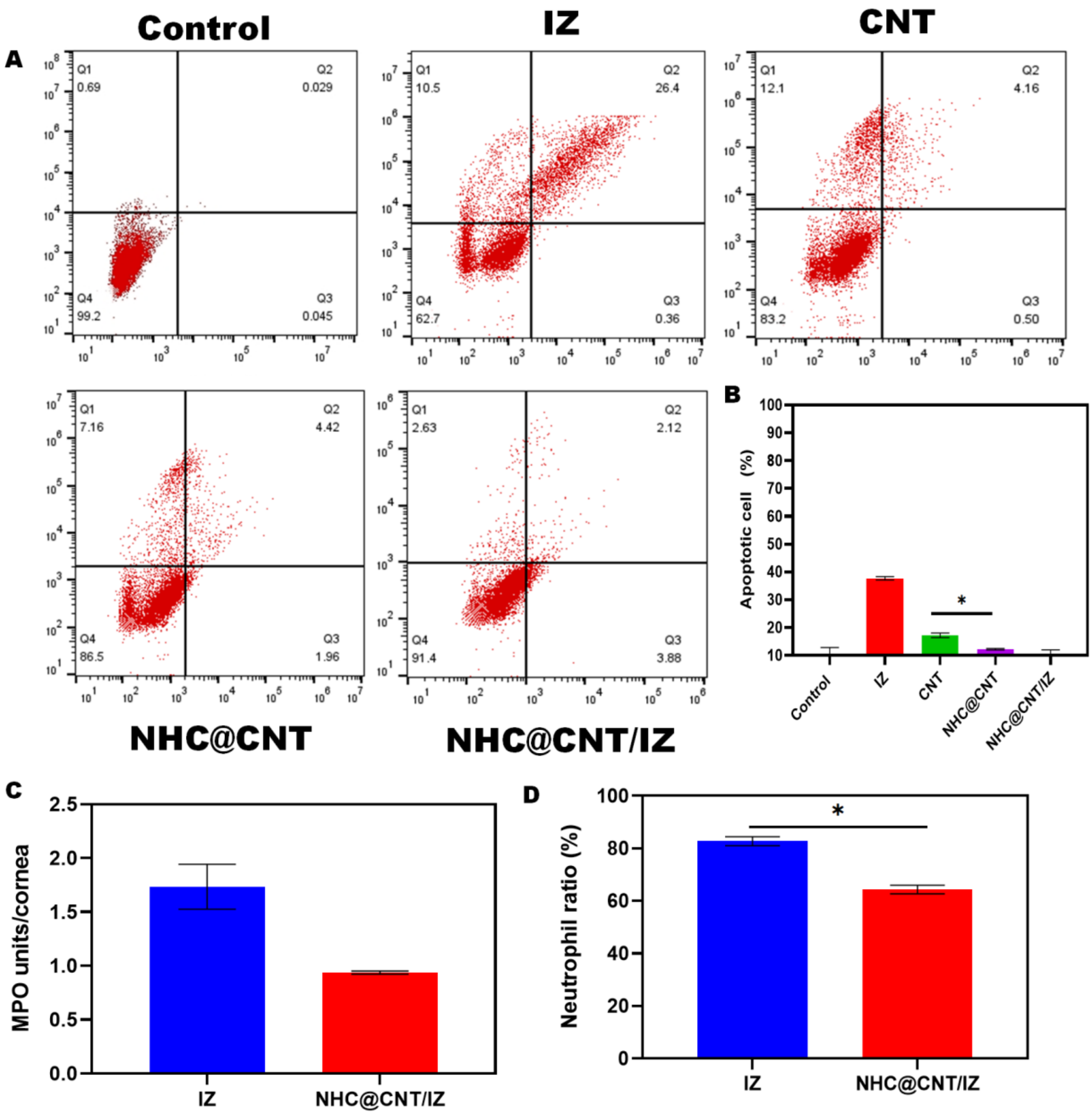

Fig S3: Effect of different materials on the production of Dectin-1 and TLR4 in C57BL/6 corneas challenged with *A. fumigatus*. Treatment with different materials significantly inhibited *A. fumigatus*-induced raising of relative gene expression with Nrf-2, IL-1β, TNF-α, IL-8, IL-6 compared with control group respectively. Treatment groups are compared with control Asterix indicates significance among groups *p<0.001.

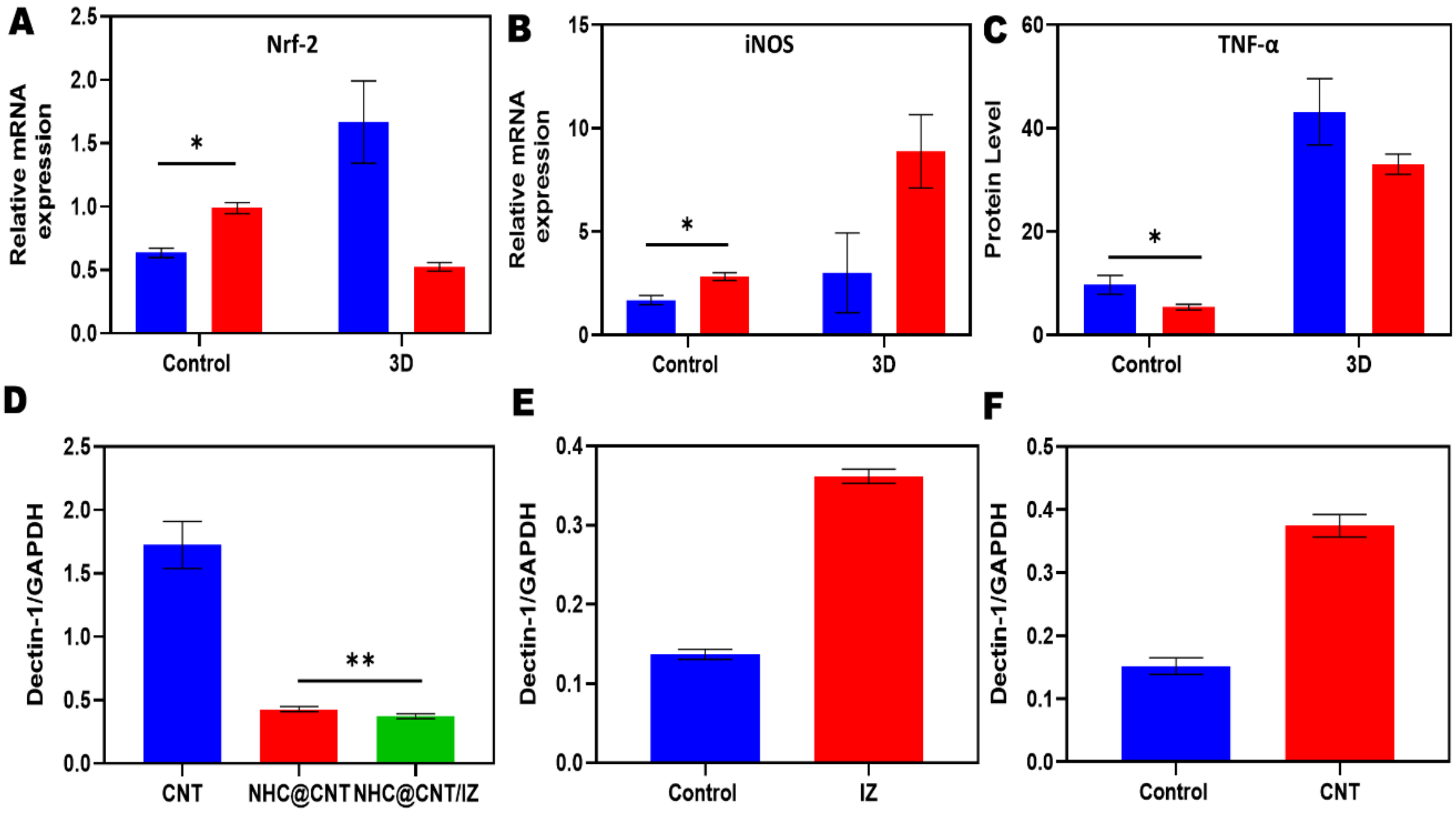

Fig S4: Relative mRNA expression of Nrf-2,iNOS,TNF-α were observed (A-C); Dectin-1/GAPDH expression of tested samples Control, IZ, CNT, NHC@CNT, NHC@CNT/IZ were expressed. All the experiments were done with triplicates and these are satistically significance between groups *p<0.001.

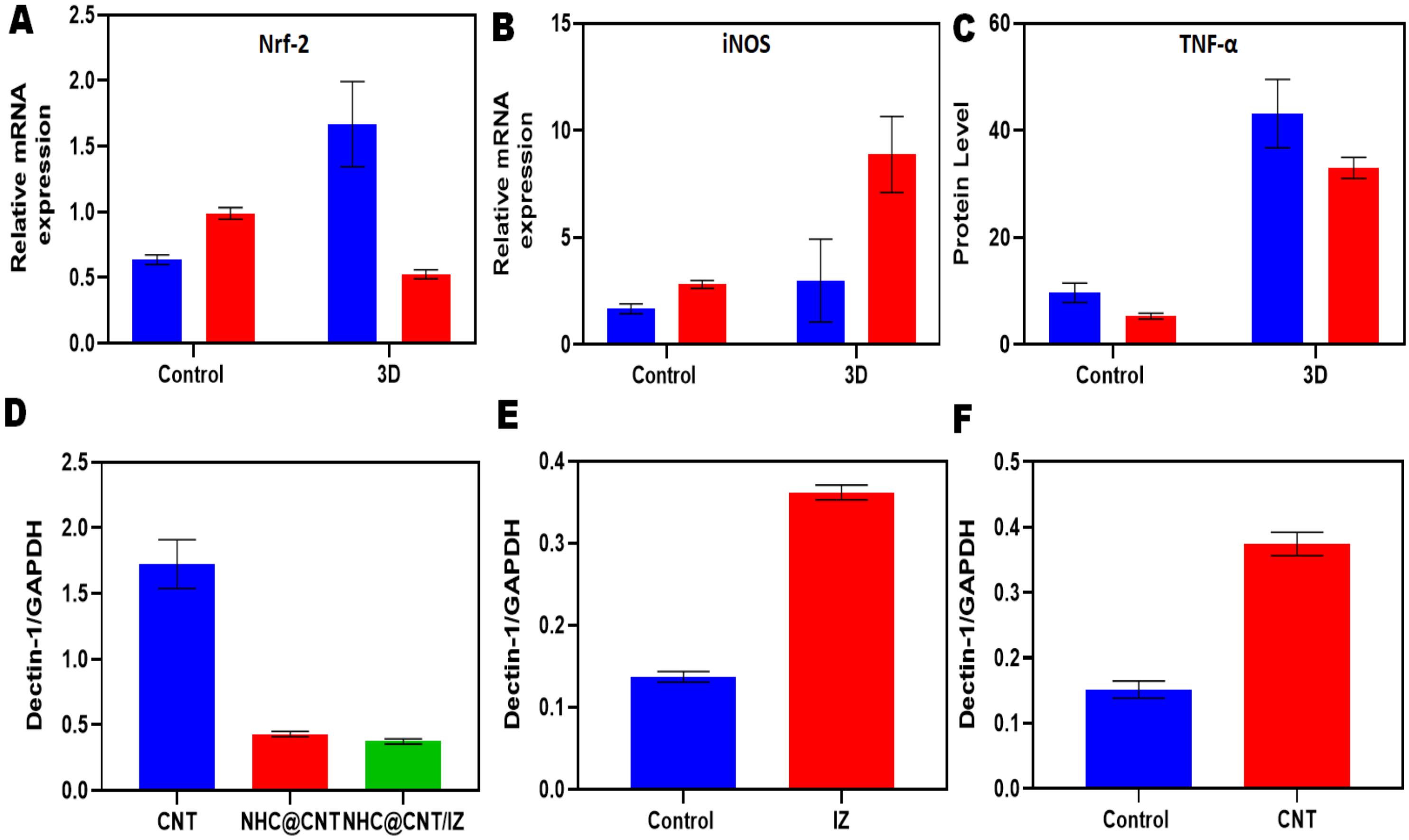

Supplement: Supplementary file 1 [file 253_2025_13529_MOESM1_ESM.pdf]
